# RefineNet‐based 2D and 3D automatic segmentations for clinical target volume and organs at risks for patients with cervical cancer in postoperative radiotherapy

**DOI:** 10.1002/acm2.13631

**Published:** 2022-05-09

**Authors:** Chengjian Xiao, Juebin Jin, Jinling Yi, Ce Han, Yongqiang Zhou, Yao Ai, Congying Xie, Xiance Jin

**Affiliations:** ^1^ Department of Radiotherapy Center Wenzhou Medical University First Affiliated Hospital Wenzhou People's Republic of China; ^2^ Department of Medical Engineering Wenzhou Medical University First Affiliated Hospital Wenzhou People's Republic of China; ^3^ Department of Radiation and Medical Oncology Wenzhou Medical University Second Affiliated Hospital Wenzhou People's Republic of China; ^4^ School of Basic Medical Science Wenzhou Medical University Wenzhou People's Republic of China

**Keywords:** automatic segmentation, cervical cancer, clinical target volume, deep learning, organs at risk

## Abstract

**Purpose:**

An accurate and reliable target volume delineation is critical for the safe and successful radiotherapy. The purpose of this study is to develop new 2D and 3D automatic segmentation models based on RefineNet for clinical target volume (CTV) and organs at risk (OARs) for postoperative cervical cancer based on computed tomography (CT) images.

**Methods:**

A 2D RefineNet and 3D RefineNetPlus3D were adapted and built to automatically segment CTVs and OARs on a total of 44 222 CT slices of 313 patients with stage I–III cervical cancer. Fully convolutional networks (FCNs), U‐Net, context encoder network (CE‐Net), UNet3D, and ResUNet3D were also trained and tested with randomly divided training and validation sets, respectively. The performances of these automatic segmentation models were evaluated by Dice similarity coefficient (DSC), Jaccard similarity coefficient, and average symmetric surface distance when comparing them with manual segmentations with the test data.

**Results:**

The DSC for RefineNet, FCN, U‐Net, CE‐Net, UNet3D, ResUNet3D, and RefineNet3D were 0.82, 0.80, 0.82, 0.81, 0.80, 0.81, and 0.82 with a mean contouring time of 3.2, 3.4, 8.2, 3.9, 9.8, 11.4, and 6.4 s, respectively. The generated RefineNetPlus3D demonstrated a good performance in the automatic segmentation of bladder, small intestine, rectum, right and left femoral heads with a DSC of 0.97, 0.95, 091, 0.98, and 0.98, respectively, with a mean computation time of 6.6 s.

**Conclusions:**

The newly adapted RefineNet and developed RefineNetPlus3D were promising automatic segmentation models with accurate and clinically acceptable CTV and OARs for cervical cancer patients in postoperative radiotherapy.

## 1 INTRODUCTION

Cervical cancer is one of the most common gynecological malignancies and the second most prevalent cancer in females.^1^ Radiotherapy is one of the main treatment options for cervical cancer in both curative and adjuvant settings. With the development of intensity‐modulated radiotherapy (IMRT) and volumetric modulated arc therapy (VMAT), the irradiation to surrounding normal organs is reduced, as well as the associated acute and chronic toxicity compared with conventional 2D and 3D conformal radiotherapy.^2^, ^3^ IMRT and VMAT use numerous beam segments to modulate the beam intensity to deliver steep dose gradients and shapes to achieve conformal dose tightly to target volumes, thereby sparing the normal tissue.^3^, ^4^ Therefore, an accurate and reliable target volume delineation is critical for the safe and successful application of IMRT and VMAT in patients with cervical cancer.

There is a clear consensus regarding the clinical target volume (CTV) in radical and postoperative radiotherapy settings using IMRT and VMAT for patients with cervical cancer.^5^ Manual delineation is still the standard practice in most clinics. However, manual delineation is not only time‐consuming, but also prone to intra‐ and interobserver variations. CTV variations of up to 19‐cm differences and twofold volume differences were reported, which resulted in significant dosimetric differences during IMRT and VMAT delivery.^6^ On the other hand, with the adoption of image‐guided and adaptive radiotherapy, a fast and accurate automatic segmentation of target volumes and organs at risk (OARs) is urgently needed.

Previously, multi‐atlas‐based and hybrid techniques have been considered the state‐of‐the‐art for automatic segmentation.^7^ Atlas‐based methods used previous manually contoured targets to match the testing images^8^ and achieved reasonable accuracy on OARs segmentations, especially for head‐and‐neck cancer patients.^9^ However, it relies heavily on the accuracy of deformable image registration and selected atlases and requires significant manual edition.^10^, ^11^ On the other hand, CTV contouring for cervical cancer is different from OARs as CTV contains the gross tumor and subclinical malignant regions with unclear boundaries, which is heavily depending on the clinical experiences of oncologists. Torheim et al. used a machine learning method (Fisher's linear discriminant analysis) to contour cervical cancer automatically based on MRI images and achieved better results compared to each individual classifier models.^12^ However, handcrafted features are required for machine learning–based methods and may not be robust for varying image appearances.^13^

With the development and wide application of deep learning, deep learning–based automatic segmentation has shown a superior performance in the reduction of target volume delineation variation for many tumors.^14^, ^15^, ^16^ As for cervical cancer, three paralleled convolutional neural networks (CNNs) with the same architecture trained following different image preprocessing methods had been applied.^17^, ^18^ However, CNNs suffer from the problem of reducing the resolution of original images while increasing the ambiguity of object boundaries inevitably.^19^ Recently, the lightweight RefineNet was introduced to refine object detectors for autonomous driving, which generates high‐resolution semantic feature by fusing coarse high‐level features with finer grained low‐level features.^20^ The purpose of this study is to modify the RefineNet and develop a RefineNetPlus3D for the automatic segmentation of CTV and OARs for postoperative cervical cancer based on computed tomography (CT) images, as well as to investigate the accuracy of the RefineNetPlus3D‐based automatic segmentation algorithm by comparing it with several other deep learning methods.

## 2 MATERIALS AND METHODS

### 2.1 Patients and contours

Patients with cervical cancer under postoperative IMRT and VMAT in authors’ hospital from January 2018 to September 2020 were retrospectively reviewed in this study. All the patients were immobilized by a thermoplastic abdominal fixation device in the supine position. CT simulation was scanned from the iliac crest to the ischial tuberosities with a 16‐slice Brilliance Big Bore CT scanner (Philips Healthcare, Cleveland, OH) at 3‐mm thickness. Intravenous contrast was injected during CT scan to enhance the contrast of target volumes. CT images were transferred using the Digital Imaging and Communications in Medicine format and reconstructed using a matrix size of 512 × 512.

Manual segmentations of the CTV and OARs were delineated and verified by two senior radiation oncologists with more than 10 years of clinical experience for cervical cancer and were taken as a ground truth for the evaluation of automatic segmentations. The target contour guideline of the Radiation Therapy Oncology Group (RTOG) 0418 and its atlas on the RTOG website was followed.^21^ After the delineation, central vaginal CTV and regional nodal CTV were interpolated into a combined CTV for the sake of easy modeling of automatic segmentation.

### 2.2 Automatic 2D and 3D segmentation models

**FIGURE 1:**
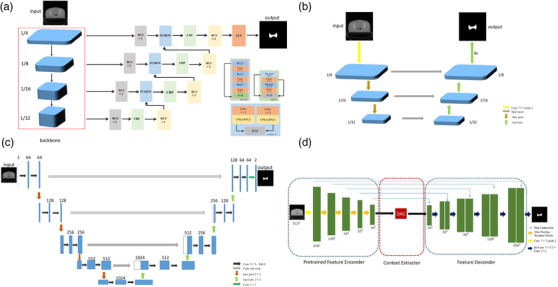
The architecture of 2D automatic segmentation models: (a) the architecture of lightweight RefineNet50; (b) the architecture of FCN; (c) the architecture of U‐Net; (d) the architecture of CE‐Net. CE‐Net, context encoder network; FCN, fully convolutional network

The adapted RefineNet in this study consists of an encoder–decoder architecture, in which the left encoding part uses a residual network (ResNet50) as a backbone network to down‐sample and extract tumor features from original images progressively, and the right decoding part consists of a residual convolutional unit (RCU), chained residual pooling (CRP), and fusion to recover the features in the final mask with the same shape as in the original images,^22^, ^23^ as shown in Figure 1a. The ResNet layers in the encoding part can be naturally divided into four blocks according to the resolution of the output feature maps. The resolution of the feature map will be reduced to one half when passing from one block to the next. Typically, the final feature map output ends up being 32 times smaller in each spatial dimension than the original image. Figure 1b–d demonstrates the encoder–decoder architectures of fully convolutional networks (FCN), U‐Net, and context encoder network (CE‐Net) for comparison.^2^, ^4^, ^5^, ^6^, ^7^, ^8^, ^9^, ^10^, ^11^, ^12^, ^13^, ^14^, ^15^, ^16^, ^17^, ^18^, ^19^, ^20^, ^21^, ^22^, ^23^, ^24^, ^25^, ^26^

**FIGURE 2:**
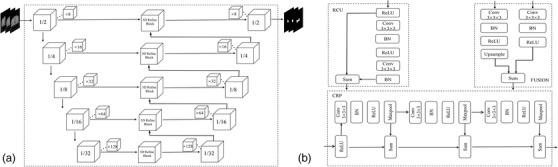
The architecture of generated 3D automatic segmentation model: (a) the architecture of RefinenetPlus3D; (b) the detail of 3D Refine block (RCU, CRP, and fusion) in the RefinenetPlus3D. CRP, chained residual pooling; RCU, residual convolutional unit

In order to use the layer thickness information more efficiently for 3D medical images, a 3D automatic segmentation model, RefineNetPlus3D, was developed based on the 2D RefineNet model mentioned earlier with all 2D operations replaced with their corresponding 3D counterparts. In the RefineNetPlus3D, the encoder part aggregates semantic information by reducing spatial information to learn features from part to whole. The decoder part receives semantic information from the bottom. We replaced the whole RefineNet decoder part with the 3D Refine block. It combines the RCU, CRP, and fusion block. In the 3D Refine block, many ReLU activations and batch normalization were added to solve the problem of gradient vanishing in the RCU, CRP, and fusion. Additionally, the first layer of down‐ and up‐sampling layers was modified to a rate of 1/2 to decrease the feature loss problem. The RefineNetPlus3D has a shortcut connection that transfers low‐level features from the encoder to the decoder and proposes an efficient and generic way of fusing coarse high‐level features (rich semantic information for classification) with finer grained low‐level features (more details information for clear boundary) to generate high‐resolution semantic features. An architecture of the RefineNetPlus3D is shown in Figure 2. UNet3D and ResUnet3D architectures were also applied in this study for the evaluation of the performance of our developed RefineNetPlus3D.^27^, ^28^

The training and testing for all the models were implemented using a GeForce RTX 2080 Ti graphics card. The training sets (which consist of CT images and manual segmentation labels) were used to tune the parameters of the networks with adopted data augmentation methods, such as random rotate, to enlarge the training sets. A weight decay of 0.8 and a learning rate policy of poly with an initial learning rate of 2e−4 for 44 training iterations and 1e−4 for 300 training iterations were applied for 2D and 3D models, respectively. The Dice‐coefficient and binary gross‐entropy loss function were used in the study for 2D and 3D models, respectively. The optimizer chose Adam that can quickly converge the network for 2D and 3D models. We chose 2 as the final batch size for the three‐dimensional network and 6 for two‐dimensional selection under computer performance constraints.

### 2.3 Model evaluation

The 2D and 3D models for CTV and OARs were trained and validated with randomly divided training and validation cohorts. Dice similarity coefficient (DSC), Jaccard similarity coefficient (JSC), and average symmetric surface distance (ASSD) were applied to evaluate the performance of automatic models by comparing them with manual segmentations in the test data sets.

The DSC is defined as

(1) $$\text{Dice}\;\text{similarity}\;\text{coefficient}=\frac{2\left|V_{\mathrm{pre}}\cap V_{\mathrm{GT}}\right|}{\left|V_{\mathrm{pre}}\right|+\left|V_{\mathrm{GT}}\right|}$$

where *V*_pre_ represents the region of interest (ROI) automatically contoured by the deep learning algorithm, and *V*_GT_ represents the ground truth ROI created by the oncologist. A value of 1 indicates a perfect concordance between two contours. ASSD is the average symmetric surface distance from points on the boundary of prediction to the boundary of ground truth and from points on the boundary of ground truth to the boundary of prediction^29^:

(2) $$\begin{array}{ccc} & & \mathrm{ASSD}=\frac{1}{\left|S\left(A\right)\right|+\left|S\left(B\right)\right|}\\ & & \;\left(\sum_{A\varepsilon S\left(A\right)}\mathrm{mi}n_{B\varepsilon S\left(B\right)}d\left(S_{A},S\left(B\right)\right)+\sum_{B\varepsilon S\left(B\right)}\mathrm{mi}n_{A\varepsilon S\left(A\right)}d\left(S_{B},S\left(A\right)\right)\right) \end{array}$$

where *A* and *B* were the surface voxels. An ASSD value of 0 mm indicates perfect segmentation. The JSC is used to compare the similarities and differences between limited sample sets. The larger the JSC value, the higher the sample similarity^30^:

(3) $$\mathrm{Jaccard}\left(A,B\right)=\frac{\left|A\cap B\right|}{\left|A\cup B\right|}$$

where *A* represents the ground truth, and *B* represents the predictive image.

### 2.4 Statistical analysis

The models were built using Pytorch1.5.0, Keras 2.4.0 and Python 3.7. The characteristics of patients were analyzed using Fisher's exact test and the Mann–Whitney *U*‐test. Statistical analyses were performed using SPSS version 19.0 (SPSS, Inc. IBM, Armonk, NY, USA) with a *p* < 0.05 considered to be statistically significant.

## 3 RESULTS

A total of 313 patients at a median age of 55 years old (range 21–80 years) with stage I–III cervical cancer were enrolled in this study. Patients were randomly divided into a training (251 patients) and validation set (31 patients) and a testing set (31 patients), respectively, with a total of 44 222 CT slices. Most patients were diagnosed as squamous cell carcinoma. Detailed characteristics of enrolled patients are shown in Table 1.

**TABLE 1:** Clinical characteristics of enrolled patients and images

	Data sets			
Characteristic	Training sets	Validation sets	Testing sets	*p*
Total number	251	31	31	
Age				0.001
Mean	54.08	55.03	53.47	
Median	55	55	53	
Range	21–78	27–80	21–78	
SD	10.98	10.59	8.80	
Slice number	35 324	4394	4504	
Histological type				0.21
Squamous cell carcinoma	209	24	26	
Adenocarcinoma	22	7	4	
Adenosquamous carcinoma	7	0	0	
Unknown	13	0	1	
Clinical stage				0.26
I	137	19	23	
II	112	12	8	
III	2	0	0	

*p* Value is calculated from the univariate association test between subgroups. Mann–Whitney *U*‐test for continues variables, Fisher's exact test for categorized variables.

**FIGURE 3:**
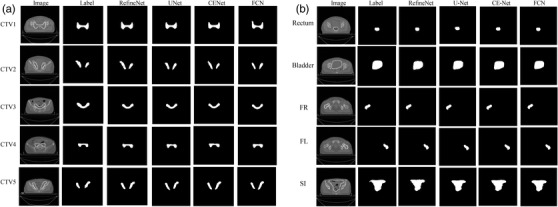
Typical automatic delineation results from 2D models: (a) clinical target volume contours in comparison with manual contours; (b) automatic delineation results of organs at risks in comparison with manual contours

Figure 3 shows the performance of 2D automatic segmentation models in comparison with manual contours for the CTVs and OARs. Quantitative evaluation among four 2D models is shown in Table 2. The DSC for RefineNet, FCN, U‐Net, and CE‐Net for CTV contouring were 0.82, 0.80, 0.82, and 0.81 with a mean contouring time for these four models being 3.2, 3.4, 8.2, and 3.9 s respectively. The mean computing time of RefineNet, FCN, U‐Net, and CE‐Net for these OARs was around 3.9, 8.2, 4.8, and 4.7 s, respectively.

**TABLE 2:** Performance evaluations of 2D automatic segmentation models for CTV and OARs

Parameters	OARs/models	RefineNet	U‐Net	CE‐Net	FCN
JSC	CTV	0.72	0.71	0.70	0.68
	Bladder	0.92	0.91	0.91	0.92
	SI	0.85	0.86	0.86	0.86
	FR	0.95	0.95	0.95	0.94
	FL	0.95	0.94	0.95	0.94
	Rectum	0.83	0.81	0.82	0.82
DSC	CTV	0.82	0.82	0.81	0.80
	Bladder	0.95	0.95	0.94	0.96
	SI	0.90	0.90	0.91	0.91
	FR	0.97	0.97	0.97	0.97
	FL	0.97	0.96	0.97	0.97
	Rectum	0.88	0.87	0.89	0.88
ASSD	CTV	4.17	4.18	4.30	4.58
	Bladder	1.24	1.28	1.34	1.29
	SI	2.64	2.44	2.42	2.59
	FR	0.54	0.49	0.50	0.57
	FL	0.49	0.48	0.49	0.50
	Rectum	1.27	1.61	1.48	1.31
Contouring time (s)	CTV	3.2	8.2	3.9	3.4
	Bladder	3.9	8.3	3.8	3.8
	SI	3.9	8.2	3.6	4.1
	FR	3.9	8.2	4.2	3.6
	FL	3.9	8.1	3.8	4.1
	Rectum	3.9	8.0	3.9	3.3

Abbreviations: ASSD, average symmetric surface distance; CE‐Net, context encoder network; CTV, clinical target volumes; DSC, Dice similarity coefficient; FCN, fully convolutional network; FL, left femoral head; FR, right femoral head; JSC, Jaccard similarity coefficient; OARs, organs at risk; SI: small intestine.

**FIGURE 4:**
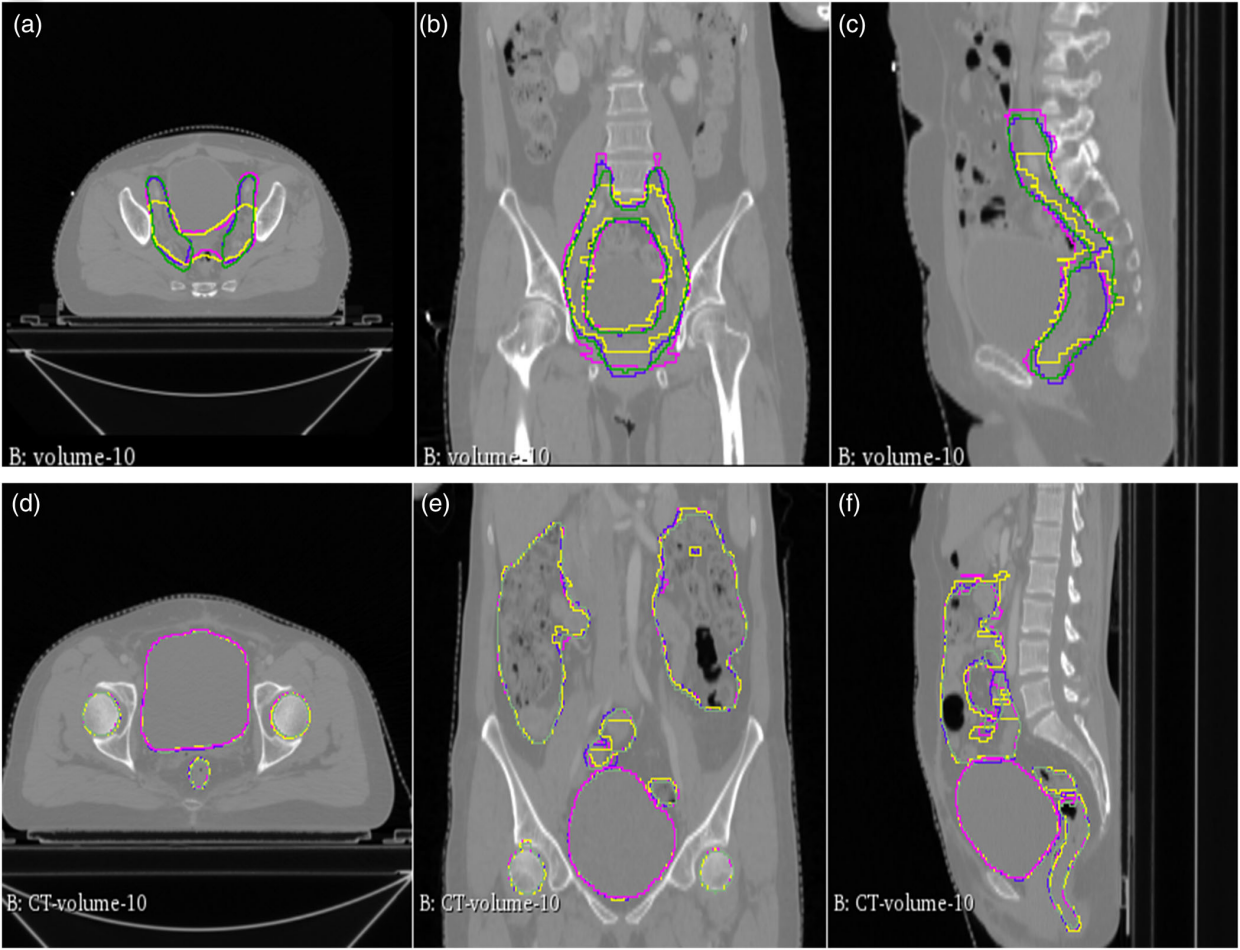
Typical automatic delineation results from 3D models: (a)–(c) clinical target volumes in axial, sagittal and coronal views; (d)–(f) contours of organs at risks in axial, sagittal, and coronal views, where yellow lines represent manual contours, purple for RefinenetPlus3D, blue for 3DResUNet, and green for 3DUNet contours

Figure 4 shows the performance of 3D models through the visualization of automatically segmented CTV and OARs for one case of a cervical cancer patient. Quantitative evaluation for these three 3D models is shown in Table 3. The DSC for UNet3D, ResUNet3D, and RefineNetPlus3D was 0.80, 0.81, and 0.82, respectively, and a mean contouring time for these three models was 9.8, 11.4, and 6.4 s, respectively. The generated RefineNetPlus3D demonstrated a good performance with a DSC of 0.97, 0.95, 0.91, 0.98, and 0.98 for bladder, small intestine, rectum, right and left femoral heads, respectively. The mean computing time of the RefineNetPlus3D for these OARs was around 6.6 s.

**TABLE 3:** Evaluation of 3D automatic segmentation models for CTVs and OARs

Parameters	OARs/models	UNet3D	ResUNet3D	RefineNetPlus3D
JSC	CTV	0.67	0.69	0.69
	Bladder	0.93	0.94	0.94
	SI	0.88	0.90	0.90
	FR	0.94	0.96	0.96
	FL	0.95	0.96	0.96
	Rectum	0.78	0.84	0.84
DSC	CTV	0.80	0.81	0.82
	Bladder	0.96	0.97	0.97
	SI	0.93	0.95	0.95
	FR	0.97	0.98	0.98
	FL	0.97	0.98	0.98
	Rectum	0.88	0.91	0.91
ASSD	CTV	3.56	3.46	2.13
	Bladder	0.59	0.48	0.30
	SI	1.68	1.45	1.02
	FR	0.34	0.23	0.16
	FL	0.29	0.20	0.15
	Rectum	1.37	0.92	0.61
Contouring time (s)	CTV	9.8	11.4	6.4
	Bladder	9.7	10.3	6.3
	SI	10.5	11.0	6.7
	FR	10.9	10.6	6.7
	FL	10.3	11.0	6.7
	Rectum	10.1	12.3	6.7

Abbreviations: ASSD, average symmetric surface distance; CTV, clinical target volumes; DSC, Dice similarity coefficient; FL, left femoral head; FR, right femoral head; JSC, Jaccard similarity coefficient; OARs, organs at risk; SI, small intestine.

## 4 DISCUSSION

Accurate and quick segmentations of target volumes and OARs are critical to the precise IMRT and VMAT optimization and delivery, as well as for the application of adaptive radiotherapy. In this study, new 2D and 3D automatic segmentation models were adapted and generated based on RefineNet for the CTV and OARs of patients with cervical cancer in postoperative radiotherapy. Both adapted 2D RefineNet and developed RefineNetPlus3D achieved a better performance in CTV segmentation and similar performance in OARs segmentation in comparison with other generally used deep learning algorithms with a shorter computing time.

During IMRT and VMAT optimization, the radiation dose is usually prescribed to tumor target volumes to achieve adequate coverage, so as to maximize tumor control and minimize radiation toxicities.^31^ However, the poorly defined tumor‐to‐normal tissue interface of cervical cancer due to the lack of tissue contrast on CT images makes CTV contouring a challenging task and results in high intra‐ and interobserver variability.^6^ Deep learning–based automatic segmentation is increasingly investigated to improve the delineation consistency and accuracy. In this study, both 2D (RefineNet, CE‐net, U‐Net, FCN) and 3D (UNet3D, ResUNet3D, RefineNetPlus3D) automatic segmentation models based on deep learning were investigated to segment automatically the CTV of cervical cancer for postoperative radiotherapy and achieved a DSC of 0.82, 0.81, 0.82, 0.80, 0.80, 0.81, and 0.82, respectively. Similarly, Ju et al. reported a DSC of 0.82 using a Dense V‐Net for the CTV delineation for cervix cancer radiotherapy.^32^ However, the DSC of our models in this study is not as good as those of CNNs CNNs in Rhee et al.,^33^ 3D CNN in Wang et al.,^34^ and 2.5 CNN networks (DpnU‐Net) in Liu^18^ with a reported DSC around 0.86 for CTV of cervical cancer. This indicated that there is a potential improvement of our adapted 2D and 3D RefineNet. Other factors that may affect the contouring accuracy need further investigation, such as image and manual contour quality.

Volume definition of OARs is a prerequisite for meaningful 3D treatment planning and for accurate dose reporting. Studies reported that the deep learning algorithm was superior to the other state‐of‐the‐art segmentation methods and commercially available software in the automatic segmentation of OARs, such as rectum and parotid.^35^ In this study, both the 2D and 3D models demonstrated a good performance in automatic segmentation for bladder, right and left femoral heads. 3D models performed a bit better than 2D models in small intestine and rectum with a mean DSC of 0.90 versus 0.95, 0.88 versus 0.91, respectively, as shown in Tables 2 and 3. As the RefineNetPlus3D developed in this study employed more high‐level feature extraction hidden layers by using RCU, CRP, and Fusion modules to aggregate contextual features, it improved the recognition of the unclear boundaries of some parts of the rectum and the small intestine.

Generally, automatic segmentation models performed better in bladder and femoral heads with DSC higher than 0.97, which has obvious contour boundaries. The relatively poor performance of these models in rectum may be due to their small volume and unclear outlines. Similarly, Elguindi et al. reported a DSC of 0.93 ± 0.04 and 0.82 ± 0.05 for bladder and rectum, respectively, using a two‐dimensional FCN and DeepLabV3+ with MRI images.^36^ Balagopal et al. also presented a similar DSC of bladder (0.95) and rectum (0.84) with deep learning–based auto‐segmentation.^37^

Saving the contouring time of radiation oncologists is an inherent product of automatic segmentation of the CTV and OARs. The average manual CTV and OAR contouring time for one cervical cancer patient was 90–120 min.^38^ In this study, the proposed algorithms took only half the computation time spent when using U‐Net under the same computer configuration. Moreover, the contouring time was only 4 s for 2D RefineNet and around 6 s for RefineNetPlus3D, respectively. On the other hand, the current results in cervical CTV and OAR contouring demonstrate that RefineNetPlus3D is able to learn high‐level semantic features well, and this method may also have the potential to be used for volume delineations in other cancers; we will explore this possibility in future studies.

The model analysis in this study was based on the whole image for segmentation prediction, not just focusing on the target area, which makes an automatic segmentation of CTV for cervical cancer more challenging. Images without target volumes acted as negative samples during modeling and affected the accuracy of the models. A good balance between positive and negative samples may further improve the performance of the models. It would also be a good exploring direction to improve the 2D and 3D models when more data were collected.

## 5 CONCLUSIONS

Deep learning–based automatic segmentation is critical for the accuracy and efficiency of radiotherapy. The newly adapted RefineNet and developed RefineNetPlus3D in this study demonstrated that it is able to learn high‐level semantic features and achieve accurate and clinically acceptable CTV and OARs automatic segmentation for cervical cancer patients in postoperative radiotherapy. The RefineNetPlus3D may also be promising for volume delineations for other cancers, which will be investigated in our future studies.

## CONFLICT OF INTEREST

The authors declare there is no conflict of interest.

## AUTHOR CONTRIBUTIONS

Conception and design: Congying Xie and Xiance Jin. Administrative support: Xiance Jin. Provision of study materials or patients: Chengjian Xiao and Juebin Jin. Collection and assembly of data: Chengjian Xiao and Juebin Jin. Data analysis and interpretation: Jinling Yi, Ce Han, Yongqiang Zhou, and Yao Ai. Manuscript writing: Chengjian Xiao, Juebin Jin, Congying Xie, and Xiance Jin. Final approval of manuscript: Congying Xie and Xiance Jin. All authors contributed to the article and approved the submitted version.
